# Laser-assisted direct roller imprinting of large-area microstructured optical surfaces

**DOI:** 10.1038/s41378-024-00650-3

**Published:** 2024-01-18

**Authors:** Keisuke Nagato, Ken Takahashi, Yuki Yajima, Masayuki Nakao

**Affiliations:** Department of Mechanical Engineering, Graduate School of Engineering, The University of Tokyo, 7-3-1 Hongo, Bunkyo-ku, Tokyo, 113-8656 Japan

**Keywords:** Micro-optics, Optical materials and structures, Engineering, Nanoscale materials

## Abstract

In this study, a high-throughput fabrication method called laser-assisted direct roller imprinting (LADRI) was developed to lower the cost of nanoimprinting large-area polymer films and to address problems associated with nanoimprinting, namely, microstructural damage and precision in flatness of entire film. With LADRI, the laser directly heats the microstructured surface of the roller mold, which heats and melts the surface of a polymethyl methacrylate (PMMA) film to replicate the microstructures on the mold rapidly. In this study, the effects of laser power density, scanning speed, size of the microstructures, and contact pressure on the replication speed were investigated experimentally. The replication speed increased as the power and scanning speed increased. However, because the film required heating until it filled the entire depth of the microstructure, an appropriate replication speed was necessary. This result was supported by simulation of the temperature distribution inside the mold and the PMMA using transient heat conduction analyses. To demonstrate the applications of LADRI, two different optical surfaces were replicated: an antireflection (AR) structure with conical structures sized several hundred nanometers and a light-extraction structure with a microlens array (MLA) comprising 10 μm lenses, for display and illumination, respectively. The replication degree of the MLA was governed by the contact pressure. Polymer flow simulation indicated that the heat conduction and flow speeds of the melted PMMA surface were comparable within several tens of micrometers. In addition, the reflectivity of the AR structure decreased from 4 to 0.5%, and the light intensity of the light-extraction structure increased by a factor of 1.47.

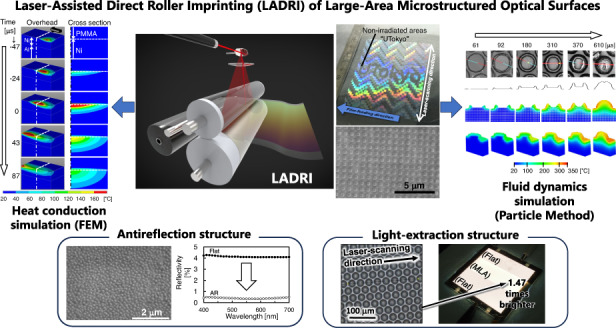

## Introduction

Polymer films with microstructures, i.e., surface structures ranging in size from less than a micrometer to several tens of micrometers, enable emerging optical functions such as antireflection^1,2^, polarization^3,4^, photonic crystallinity^5,6^, light extraction^7,8^, and light diffusivity^9,10^; therefore, they are expected to enhance the functions of various optical devices, such as displays (including holographic and illumination devices), solar cells, and traffic signal lights. Furthermore, flexible displays^11,12^, flexible sensors^13,14^, organic electroluminescent light-emitting diodes^15,16^, and flexible solar cells^17,18^ based on microstructured polymer films have recently been developed as next-generation optical devices. The microstructures can also perform novel functions in immunoassay chips^19^, cell culture plates^20^, and nanofluidic devices^21^. Such large-area applications require lower cost and higher-throughput microstructure fabrication methods compared with conventional photolithography technologies, such as ultraviolet (UV) and electron-beam (EB) lithography.

Since its development, nanoimprint lithography (NIL)^22^ has been increasingly researched as an alternative to UV or EB lithography because an NIL system is less expensive than that used in conventional lithography and does not face the diffraction limits of photolithography. UV-NIL^23,24^ is the most useful candidate for NIL, wherein a thin UV-curable polymer resist coated on Si or another flat substrate is imprinted by a nanostructured mold and then UV-cured and patterned. In UV-NIL, the limitation of miniaturization is low because polymers with small molecules are generally used, and the accuracy of the pattern position in the substrates is high because no thermal process is applied. Similarly, soft lithography^21^ replicates microstructures on thermocurable polymers and is mainly used for bioapplications because of its usability in laboratories. Thermal nanoimprinting (TNI)^25,26^ is a promising low-cost method for patterning nano- or microstructures on the surfaces of bulk polymers. TNI entails heating various thermoplastic polymers such that the melted polymer fills the nanostructures in the mold^27^. The polymer and mold are then cooled, and the mold is removed. TNI can be more rapidly applied to various thermoplastic polymers than to UV-curable or thermocurable polymers. Therefore, TNI does not require high-precision optics or a polymer coating. Similarly, unlike in photo- or EB lithography, UV-irradiation systems, as in UV-NIL, are not needed. Therefore, TNI is the most promising mass-production method for applications involving large-area microstructured films.

However, conventional TNI has several limitations. In TNI, the heaters and coolers are located inside both punches for the mold and polymer film. In this process, the punches, mold, and polymer film are all heated above the glass transition temperature (*T*_g_) of the polymer surface. Subsequently, the heat is removed using coolers that cool the polymer surface below *T*_g_. This process indicates that the reductions in the cycle time and energy consumption are limited by the thermal conductivities and heat capacities of the punches, mold, and polymer. Direct surface-heating methods have been proposed to overcome these issues. The first such method reported was the laser-assisted direct imprint (LADI) method^28^, wherein a laser is used to heat and melt the silicon substrate surface^28^ and thermoplastic resist film on a silicon substrate^29^ directly through a quartz mold, thus replicating the nanostructures on the silicon surface. Following LADI, thin-film current heating^30,31^ and laser-assisted heating^32,33^ of mold surfaces were also proposed and demonstrated to enable rapid replication of the microstructures on bulk polymer films or glass substrates.

Roller imprinting is a promising method of fabricating large-area microstructured films^34,35^ with continuity. Compared with the stamping method, roller imprinting has a low risk of partial contact because its contact area is one-dimensional, whereas that of the stamping method is two-dimensional. However, roller nanoimprinting is difficult when using TNI, as mentioned previously, because the film surface must be heated and then cooled completely below *T*_g_ while the polymer surface is in contact with the mold. If the polymer film is demolded before it is cooled, the replicated microstructures are easily damaged by the reflow phenomenon^36–38^. To overcome this issue, sheet nanoimprinting uses a belt mold^39^ to separate the heating and cooling functions of rollers and enables long-term contact; however, the belt mold is expensive, and achieving precision in flatness becomes difficult. This article proposes laser-assisted direct roller imprinting (LADRI), wherein a laser-assisted replication method with laser scanning irradiation^40^ is used for roller imprinting. The laser is irradiated onto the roller-mold surface where a polymethyl methacrylate (PMMA) film is pressed against the roller mold by the backup glass roller. The effects of the laser power density and irradiation time on the scanning speed were investigated by performing replication experiments of diffraction patterns and transient conduction analysis using a three-dimensional (3D) finite-element method (FEM). Furthermore, antireflection (AR) structures with subwavelength-size conical patterns and a light-extraction structure consisting of microlens array (MLA) patterns sized several tens of micrometers were replicated using LADRI, and their replication and optical performance were investigated. Using the MLA pattern, the effects of the heat conduction and flow phenomena were experimentally and quantitatively investigated by varying the irradiation time and pressure.

### Laser-assisted direct roller imprinting

**Fig. 1:**
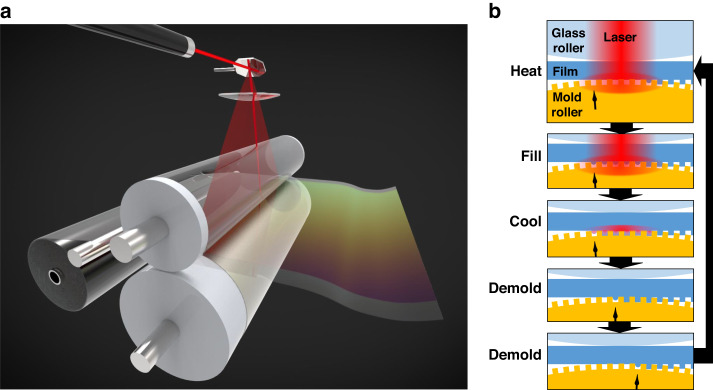
Laser-assisted direct roller imprinting (LADRI). **a** Schematic of the LADRI system. **b** Schematic of the imprinting mechanism

**Fig. 2:**
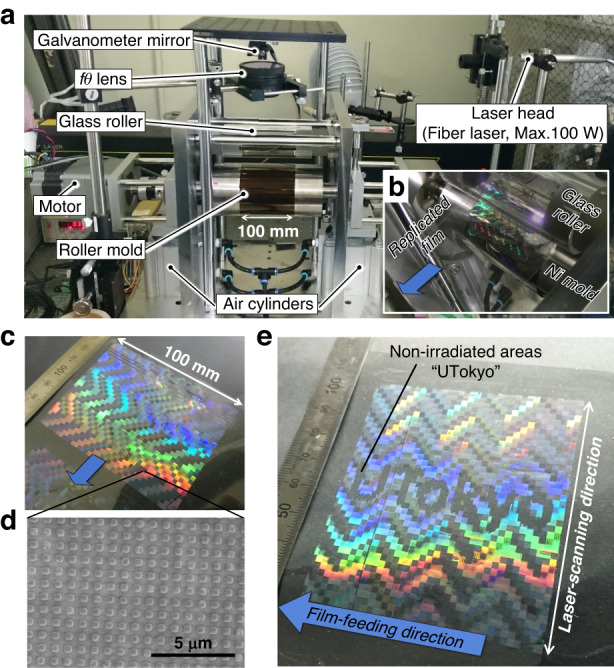
Images of the experimental setup and samples. **a** LADRI system. **b** Magnified image of the roller-imprinted area. **c** PMMA film on which the diffraction pattern is replicated. **d** SEM image of the diffraction pattern. **e** Replicated PMMA film with non-irradiated areas forming the letters “UTokyo”

Here, the concept and scheme of LADRI are described in detail. Figure 1a, b shows schematics of the LADRI system and microscopic mechanisms of heating, filling, cooling, and demolding, respectively. A roller mold with a microstructured surface and a backup glass roller were used to press the polymer film. While the rollers fed the film, a galvanometer mirror was used in combination with an *fθ* lens to direct and focus the laser onto the mold surface through the backup glass roller and polymer film. The degrees of laser absorption of the glass roller and polymer film were required to be sufficiently smaller than that of the mold surface. Figure 2a shows a photograph of the experimental setup used in this study; the laser was a 100 W single-mode fiber laser (wavelength: 1070 nm), the backup glass roller was a 50-mm-diameter quartz cylinder, the polymer film comprised a 75 μm-thick PMMA film (*T*_g_ approximately 100 °C) that was inserted beneath the glass roller and pressed against the roller mold, and the roller mold consisted of a 200-μm-thick Ni stamper wrapped around a 70-mm-diameter Al-alloy cylinder. The optics were designed considering that the laser refracted at the top surface of quartz roller. The size of the laser spot was 340 μm, as confirmed by sliding a knife edge between the quartz roller and the roller mold. The power was measured where the beam exited the backup quartz roller. A periodic hole array with pitches of 400–800 nm was fabricated on the surface of the Ni stamper with an absorption degree of approximately 29%, which was calculated by measuring the degree of laser reflection. The laser directly heated the mold surface, and it was immediately cooled via conduction into the roller mold along with the polymer substrate after the laser was moved away. Figure 2b shows a photograph of the microstructured polymer immediately after replication and feeding.

## Results and discussion

### Replication properties of LADRI

#### Experimental setup and demonstration of diffraction pattern

To investigate the performance of the replication function, diffraction patterns were repeatedly fabricated. The Ni stamper with the microstructure was prepared by electroplating a master substrate, which was prepared via EB lithography. The patterns on the mold contained a 250–500 nm pitch and an array of 230 nm square holes. Figure 2c–e shows photographs and scanning electron microscopy (SEM, SU-8010, Hitachi High-Tech Corporation) images of the Ni mold and an example of a replicated PMMA film, respectively. The color variation with a 5 mm interval is attributed to variation in the pattern pitch. The diffraction patterns were completely replicated throughout an area of 100 mm × 100 mm, as confirmed by atomic force microscopy (AFM). The laser, JL-SP0178 (SPI Lasers, single-mode fiber laser, wavelength: 1070 nm), was focused using an *fθ* lens to form an irradiation spot sized 340 μm. In this case, the power and scanning speed were 100 W and 6.1 m/s, respectively. Furthermore, we demonstrated fabrication of the pattern. Figure 2e shows a photograph of the film patterned with “UTokyo” using the shutter of the laser. The conditions were identical to those used for the samples shown in Fig. 2e.

Interestingly, the films had almost no crinkles in the replicated area or at the edges between the irradiated and non-irradiated areas. This result is in marked contrast to conventional thermal nanoimprinting, where crinkles generally form, particularly at the edge of the mold, because the entire film is heated and cooled. In contrast, in LADRI, because heating and cooling occurred only at the surface of the film, the base material of the film was maintained at ~25 °C, i.e., at room temperature, during the process. The crinkling force is exerted by the surface where heated and cooled across *T*_g_. However, for example, if the depth of heated and cooled area was 7 μm, the depth of non-heated area would be 68 μm, which implies that the difference in the second moment of area could be calculated ~1000 times. This factor is the reason that no crinkle was observed. The quality of the dimensions of nanostructures should be considered because the difference between the coefficients of thermal expansion of Ni and PMMA is large. This issue will be studied in the future. An additional advantage of LADRI is that the interior of the film is not heated because the base material is maintained at room temperature; thus, the film thickness is not reduced and the film remains flat.

#### Numerical simulation of heat conduction

Heat conduction was investigated by performing a simulation with a 3D FEM generated in ANSYS software by the CYBERNET SYSTEM. In the simulation, flat layers of Ni, PMMA, and SiO_2_ were modeled.

The heat conduction is expressed as follows:

1 $$\rho c\frac{\partial T}{\partial t}=\lambda \left(\frac{{\partial }^{2}T}{\partial {x}^{2}}+\frac{{\partial }^{2}T}{\partial {y}^{2}}+\frac{{\partial }^{2}T}{\partial {z}^{2}}\right)+Q,$$

where $\rho$ is the density; $c$ is the heat capacity; $\lambda$ is the heat conductivity, as indicated in Table 1; and *Q* [W/m^3^] is the internal heat generation (Gaussian distribution).

**Table 1:** Thermal properties of the materials used in the simulation

	Density $\rho$ [10^3^ kg/m^3^]	Heat capacity $c$ [J/kg·K]	Heat conductivity $\lambda$ [W/m·K]
Ni	8.9	440	91
SiO_2_	2.2	710	1.4
PMMA	1.2	1500	0.2

**Fig. 3:**
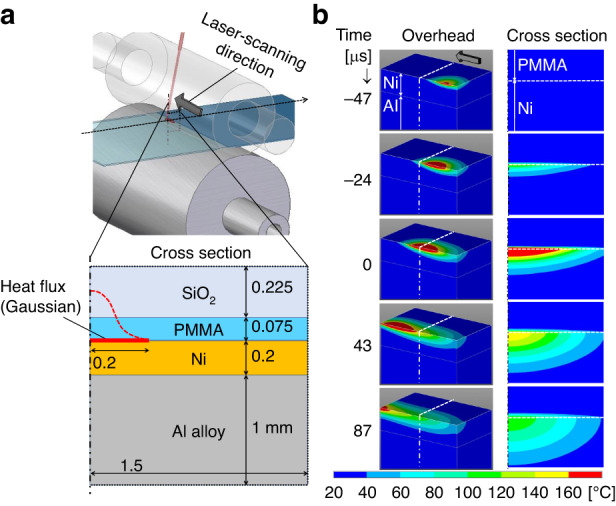
Non-steady heat conduction results. **a** 3D model used in the FEM analysis. **b** Simulated temperature distributions: overhead and cross-sectional

The heat source was located at the boundary between Ni and PMMA. The thermal contact resistances between Ni and PMMA and between PMMA and SiO_2_ were set to 0. Table 1 lists the physical properties of Ni, SiO_2_, and PMMA. Figure 3 shows the FEM model and an example of the temperature distribution calculated using FEM. The initial temperature of the entire system was set to 25 °C. Because the heat conductivity of Ni is higher than that of PMMA, the heat generated at the Ni surface flows toward the interior of Ni. As depicted by these contours, the ratio of the area with a temperature higher than *T*_g_ in the depth direction is smaller for shorter irradiation times. This trend indicates that using a shorter irradiation time is more efficient for heating the PMMA surface to *T*_g_. The mold temperature increases until reaching thermal equilibrium when the replication is continued; this effect will be studied in the future, as this study is limited to demonstration. Nonetheless, the experimental setup should be designed with continuous cooling, for example, by using a cooling medium inside of the rollers.

**Fig. 4:**
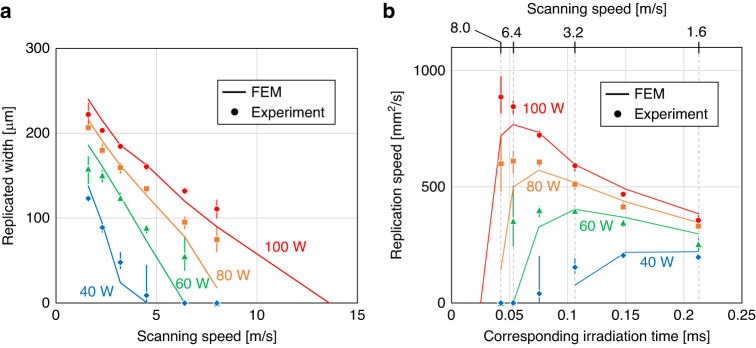
FEM calculation and experimental results for various power levels and irradiation times. **a** Replicated width as a function of scanning speed. **b** Replication speed as a function of irradiation time

The replicated width obtained by the FEM and that measured in the experiments are plotted in Fig. 4a as functions of the scanning speed. The replication speeds are plotted in Fig. 4b as functions of the irradiation time corresponding to the scanning speed. The irradiation time was determined using the following process. Assuming that “replication by laser scanning” comprises a series of replications in which the laser irradiates the target points, the irradiation time (*t*_spot_) can be approximately determined from the laser diameter (${d}_{{\rm{spot}}}$) and scanning speed (${v}_{{\rm{scan}}}$) as follows:

2 $${t}_{{\rm{spot}}}=\frac{{d}_{{\rm{spot}}}}{{v}_{{\rm{scan}}}}.$$

In particular, because the laser diameter was 0.34 mm, the irradiation time corresponding to a scanning speed of 3.2 m/s was calculated to be 0.106 ms.

In the FEM, the replication speed (${u}_{{\rm{rep}}}$) is determined by the width of the area (${w}_{{\rm{rep}}}$):

3 $${u}_{{\rm{rep}}}={w}_{{\rm{rep}}}\cdot {v}_{{\rm{scan}}},$$

when the temperature exceeds 100 °C. The heat transfer coefficient (HTC) was set to 65,000 W/m^2^K such that the experimental results agreed with the simulation results for laser power and scan speed settings of 100 W and 8 m/s, respectively. The absorption was measured as 29%, as described previously, and the internal heating was set to 29 W as a boundary condition. The HTC must increase during filling because the effective contact area increases. However, the simulation and experimental observation of the polymer and the measurement of the HTC during the filling process are difficult. Therefore, we fixed the HTC value in the simulations.

Experimentally, the replication speed was determined based on the width of the replicated area. The observed trends in the FEM and experimental results are similar, indicating that the contact thermal resistance can be determined from the contact area between the mold and polymer surface. The physical phenomena associated with the polymer during the filling of the mold are complex because the values of many of the parameters of the model are unknown, such as the dependence of the viscosity on the temperature around *T*_g_ and shear stress, surface tension against the mold and air, and dependence of laser absorption on the filling degree. Therefore, understanding these dynamic and transient phenomena at the microscale will be a focus of subsequent studies. As shown in Fig. 4b, the highest replication speed was reported for each power level. At higher scanning speeds, the time needed to heat the surface area to a certain heat capacity and increase its temperature to *T*_g_ becomes dominant. As an alternative perspective to the actual phenomena in the experiment, the fluidization of the polymer around the surface and filling of the mold are the longest processes that occur at higher scanning speeds.

### Replication of optical applications

#### Antireflection structure

**Fig. 5:**
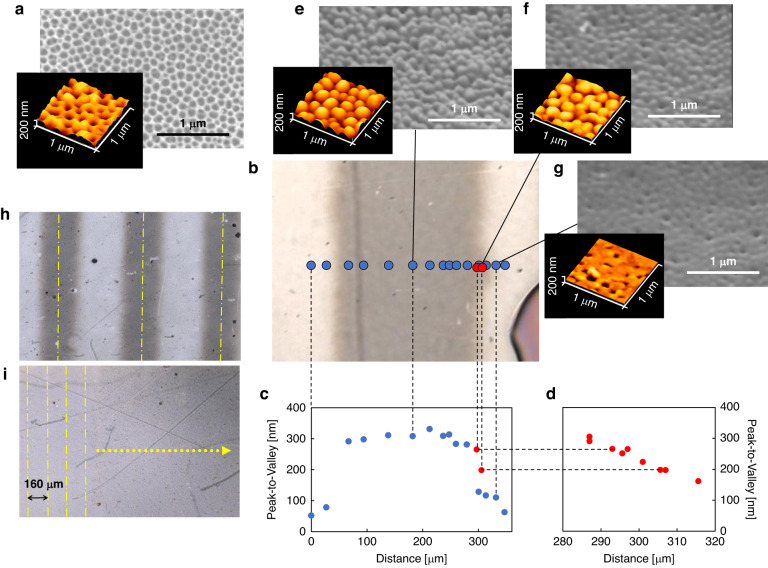
Replicated AR structures. **a** SEM images and AFM profiles of the AR-structured mold. **b** Optical microscope image of the replicated PMMA film. **c** Plots of peak-to-valley structures across the laser trajectory direction. **d** Magnified plots of the peak-to-valley structures. AFM profiles **e** at the center, **f** at the edge, and **g** far from the edge of the scanning trajectory. Optical microscopy images of the area replicated by scanning laser with **h** wide and **i** narrow intervals

A subwavelength-sized conical structure can be converted into an AR surface for a clearer display^30^. The replication of the AR structure using LADRI is presented here. Figure 5a shows a top-view SEM image of the Ni mold, which was fabricated via Ni electroplating using a master replica. The master replica was prepared using UV-NIL spectroscopy and a mold consisting of anodic alumina nanoholes^41^. The average height and pitch of the holes in the Ni mold were 300 and 120 nm, respectively. The LADRI conditions were the same as those used to fabricate the diffraction pattern. Figure 5b shows an optical microscope image of the surface, which was replicated by a single laser scan. The structures replicated through the patterned and unpatterned areas were investigated using AFM. The dark area at the bottom right of the figure is a marker for determining the positions measured using optical microscopy, AFM, and SEM. The heights are plotted in Fig. 5c. The dark area is ~250 μm wide (those in Fig. 5b were almost completely replicated). The area around the edges between the dark and bright areas (Fig. 5b) was inspected in detail, as shown in Fig. 5d. The area with an edge length of 30 μm exhibits a slight gradient. In the area outside this edge, an ~100 nm pattern is replicated. These areas may have been heated via conduction in the plane direction; however, the heat-conducting time was too short to allow the polymer to flow. The AFM profiles and SEM images at the center, edge, and outside the edge are shown in Fig. 5e, f, and g, respectively. Figure 5h, i presents optical microscope images of the replicated area at 600 and 160 μm intervals, respectively. Figure 5i reveals no seam in the area that was scanned twice, as also confirmed via SEM observation. The length of the contact area was greater than 1 mm, as confirmed by a pressure-sensitive paper.

**Fig. 6:**
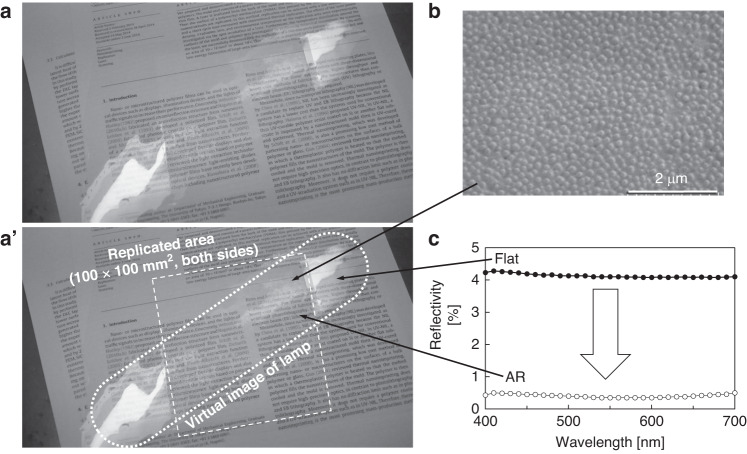
Antireflection structure replication results. **a**, **a’** Photographs of the virtual images of a straight luminescence lamp on the PMMA film without and with AR on both sides, respectively, against a document. **b** SEM image of AR. **c** Reflectivity of the AR structure on one side of the PMMA film

Based on this constraint, a large-area AR structure can be replicated. Figure 6a shows a photograph of a PMMA sheet, where a 100 mm × 100 mm area was replicated on both sides and placed on a document and a virtual image of a straight lamp was reflected. The lamp appears thin only in the replicated area. Figure 6b depicts an SEM image of the replicated area. Figure 6c presents the reflectivity over the visible wavelength range, measured using a spectrophotometer (CM-2600d, Konica Minolta Sensing, Inc.). When measuring the reflectivity of one side, the reverse side of the PMMA film was attached to a black PMMA plate by using double-sided tape with the same refractive index as PMMA^2^. In this study, the reflectivity on a flat surface was ~4%, which can be modeled by the Fresnel reflection *R*_Fresnel_ as follows:

4 $${R}_{{\rm{Fresnel}}}={\left(\frac{{n}_{{\rm{air}}}-{n}_{{\rm{PMMA}}}}{{n}_{{\rm{air}}}+{n}_{{\rm{PMMA}}}}\right)}^{2},$$

where the refractive index of PMMA, *n*_PMMA_, is ~1.5 and that of air is 1.0. In contrast, the reflectivity of the preliminary area was below 0.5%. Notably, because the film shown in Fig. 6a has AR structures on both sides, the virtual image reflects 1% reflectivity AR surfaces and 8% reflectivity flat surfaces.

As demonstrated the experimental results of the diffraction pattern, LADRI has the advantages of high-speed replication, no residual stress inside the film due to thermal damage, and large-area optical applications.

#### Light-extraction structure

**Fig. 7:**
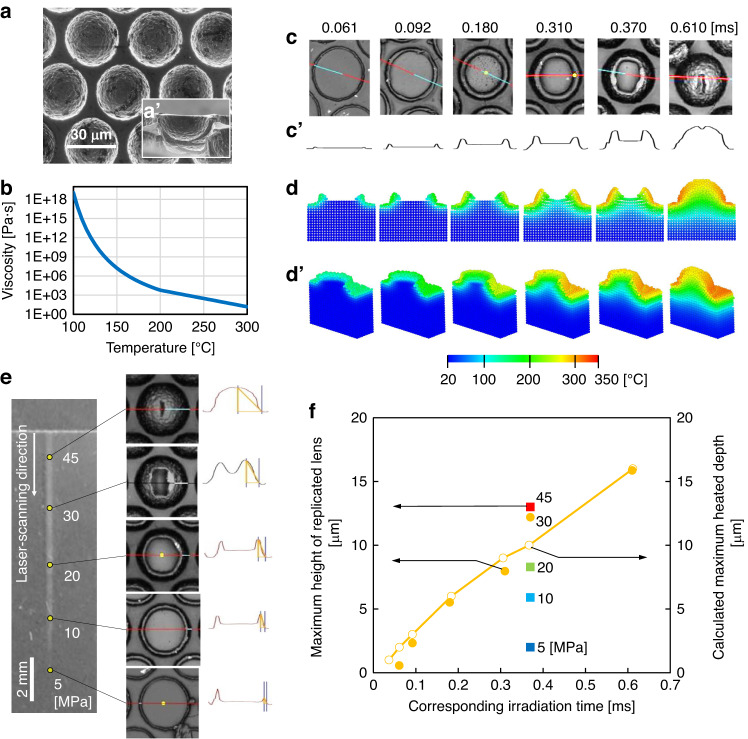
MLA replication results. **a** Top and **a’** cross-sectional SEM images of the MLA mold. **b** Temperature-dependent polymer viscosity applied to the simulation. **c** Top images and **c’** profiles of MLA with various scanning speeds corresponding to the irradiation time obtained via laser microscopy. **d** Cross-sections and **d’** top views of MLA simulated by the particle method. **e** Laser-microscopy images of the pattern under various pressures. **f** Maximum height of the replicated MLA and calculated maximum heated depth as a function of irradiation time

In the second application, a light-extraction structure^42^ was replicated using LADRI. The resulting mold contained an array of half-spherical lenses, each with a diameter of 30 μm and a height of 15 μm (SinkLab. Ltd.), as depicted in Fig. 7a. Using LADRI for this size, replication could be stopped by varying the scanning speed. Figure 7b shows the temperature-dependent polymer viscosity applied in the simulation. Figure 7c presents the optical microscopy images and surface profiles of the replicated structures obtained by a confocal laser microscope (LEXT OLS4100, OLYMPUS, Ltd.), respectively. To investigate the heat conduction, decrease in viscosity, and flow among the mold surfaces, a thermal fluid simulation was performed according to our previous study^43^. The Particle Method (ParticleWorks, Prometech Software, Inc.) was used to represent time-dependent surface deformation in the experiment, as shown in Figs. 7d and 7d’. The temperature was increased only in areas where the scale was smaller than the size of the microstructure because the rate of heat conduction is lower than the rate at which the viscosity decreases. This dynamic was investigated from the perspective of the boundary separating the temperatures higher and lower than 100 °C, which corresponds to *T*_g_. This trend indicates that the heat conduction is slower than the polymer flow. However, for the nanostructures, the heat is conducted faster than the polymer flow, as shown in Fig. 5. This finding demonstrates the design principle by which the laser parameters should be determined considering the microstructure size.

We also investigated the effect of pressure on the degree of replication using a combinatorial method, as shown in Fig. 7e. The pressure distribution was determined by the gradient of the pressing forces at the cylinder bearings, and the pressure was measured using a pressure sensor. The height of the replicated lens increases with the flow. Figure 7f presents the maximum height of the replicated lens as functions of the irradiation time and pressure with filled spheres. In the graph obtained via FEM, the depths to which heating to 100 °C is possible are indicated with hollow spheres. The calculated depth corresponds to the height of the replicated lens determined experimentally at 30 MPa. At pressures lower than 30 MPa, the polymer flow pressure is insufficient, and the height is lower than that at 30 MPa. This tendency agrees with the result of our previous study, in which a rectangular structure was used^44^.

**Fig. 8:**
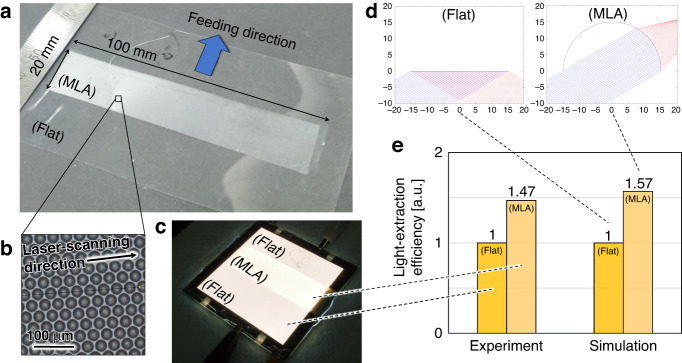
Demonstration of light extraction of organic electroluminescence by replicated MLA film. **a** Photograph of the film with MLA. **b** Magnified optical microscope image of the MLA. **c** Illuminated organic electroluminescence on which the MLA film is placed. **d** Simulated light rays with and without MLA. **e** Light-extraction efficiencies of the MLA surface compared with a flat surface in the experiment and simulation

The optical properties of the replicated MLAs were thus investigated. Figure 8a shows a photograph of the PMMA film with the MLA replicated by LADRI, as shown in Fig. 8b, for a length of 20 mm. The film was placed on an organic electroluminescence illumination substrate (OLED-010P, Konica Minolta, Inc.) illuminated at 5 V and 0.5 A. To match the refractive index of the clearance, glycerin (refractive index: 1.47) was added. Figure 8d shows an image of light extraction by the MLA. The light-extraction efficiency was measured using an omnidirectional angle-resolved scattered light measurement method. The light extraction was enhanced by the MLA to 1.47 times that with the flat surface. To determine this value, the light extracted from inside the polymer was simulated using the trajectory method^33^. Figure 8e shows an example result wherein light is reflected on the flat surface and extracted at the surface of the microlens. The simulated extraction efficiency was 1.57. The simulation model had only one lens, and the light extracted from the bottom of the lens was recorded. However, in the experiment, light from the bottom of the lens coupled onto the neighboring lens, which caused the simulated light-extraction efficiency to be 10% higher than that in the experiment. This methodology can be applied to design structures such that further light extraction can be realized.

## Conclusion

We devised LADRI, a new replication method for large-area microstructures, combining the concepts of LADI and roller imprinting. The replication properties depending on laser power and scanning speed were investigated by performing replication experiments and FEM thermal simulation using diffraction patterns. The simulation results agreed with the experimental observations, and the high efficiency of LADRI was successfully demonstrated. We also found that a 75-μm-thick PMMA film exhibited no crinkles because only the surface was heated and cooled. The quantitative investigation of locally raised temperatures and residual stress due to the difference between the coefficient of thermal expansion of Ni mold and that of PMMA film. We further demonstrated the enhancement in the optical performances of two more optical applications; antireflection and light-extraction structures. In addition, we clarified the fluid dynamics of polymer and pressure dependence on the degree of replication in LADRI replication of sub-10-μm structures.
